# Applying the cold plasma dispersion relation to whistler mode chorus waves: EMFISIS wave measurements from the Van Allen Probes

**DOI:** 10.1002/2014JA020808

**Published:** 2015-02-17

**Authors:** D P Hartley, Y Chen, C A Kletzing, M H Denton, W S Kurth

**Affiliations:** 1 Physics Department, Lancaster UniversityLancaster, UK; 2 Los Alamos National LaboratoryLos Alamos, New Mexico, USA; 3 Department of Physics and Astronomy, University of IowaIowa City, Iowa, USA; 4 Space Science InstituteBoulder, Colorado, USA

**Keywords:** Van Allen Probes, EMFISIS, chorus waves, wave-particle interactions, radiation belts, energetic electrons

## Abstract

Most theoretical wave models require the power in the wave magnetic field in order to determine the effect of chorus waves on radiation belt electrons. However, researchers typically use the cold plasma dispersion relation to approximate the magnetic wave power when only electric field data are available. In this study, the validity of using the cold plasma dispersion relation in this context is tested using Electric and Magnetic Field Instrument Suite and Integrated Science (EMFISIS) observations of both the electric and magnetic spectral intensities in the chorus wave band (0.1–0.9 *f*_ce_). Results from this study indicate that the calculated wave intensity is least accurate during periods of enhanced wave activity. For observed wave intensities >10^−3^ nT^2^, using the cold plasma dispersion relation results in an underestimate of the wave intensity by a factor of 2 or greater 56% of the time over the full chorus wave band, 60% of the time for lower band chorus, and 59% of the time for upper band chorus. Hence, during active periods, empirical chorus wave models that are reliant on the cold plasma dispersion relation will underestimate chorus wave intensities to a significant degree, thus causing questionable calculation of wave-particle resonance effects on MeV electrons.

## 1. Introduction

Relativistic electron fluxes in the outer radiation belt (3 < L < 7) are highly dynamic during periods with enhanced geomagnetic activity [e.g., *Onsager et al.*, 2002; *Green et al.*, 2004; *Borovsky and Denton*, 2009; *Hartley et al.*, 2014]. This variability is driven by an imbalance between source, loss, and transport mechanisms, each of which may become enhanced during geomagnetic storms [e.g., *Reeves et al.*, 2003; *Chen et al.*, 2007a; *Liemohn and Chan*, 2007; *Morley et al.*, 2010; *Turner et al.*, 2012; *Hartley et al.*, 2013].

Previous studies indicate that enhancements of energetic electrons in the outer radiation belt are driven by acceleration processes within the magnetosphere [e.g., *Li et al.*, 1997]. It is well established that radial diffusion, which violates the third adiabatic invariant of particle motion, can transport electrons inward and therefore increase their energy through betatron and Fermi acceleration processes [e.g., *Schulz and Lanzerotti*, 1974]. This type of acceleration requires positive gradients in the phase space density versus L shell profile inward of the source region. Recent studies have shown that persistent local maxima in the phase space density profiles exist between L = 4 and L = 5.5 [e.g., *Green and Kivelson*, 2004; *Chen et al.*, 2007b; *Turner et al.*, 2010; *Shprits et al.*, 2012; *Reeves et al.*, 2013]. These studies demonstrate that local acceleration mechanisms, such as wave-particle interactions, which violate the first and second adiabatic invariants of particle motion, are also at work within the outer belt region.

Whistler mode chorus waves are considered to be highly efficient for local acceleration of electrons up to relativistic energies [*Summers et al.*, 1998; *Thorne*, 2010]. Chorus waves are in the VLF range (∼100 Hz to tens of kilohertz) and typically occur in two bands, lower and upper, typically separated by a gap at half of the electron gyrofrequency. Modeling electron resonances with this type of wave requires the knowledge of chorus distributions, a topic that has been actively studied [e.g., *Horne and Thorne*, 2003; *Meredith et al.*, 2003a; *Li et al.*, [Bibr b15],[Bibr b16]; *Tu et al.*, 2013]. Aside from the recently developed particle proxies [e.g., *Li et al.*, 2013; *Chen et al.*, 2014], empirical chorus models [e.g., *Meredith et al.*, [Bibr b20],[Bibr b23]] are traditionally used. However, when no magnetic wave field data were available, previous studies used cold plasma theory to infer the magnetic wave power from electric field observations, and thus, current empirical wave models are based on this assumption. Recently, equipped with comprehensive wave and plasma instruments, the successfully operating Van Allen Probes mission provides an opportunity to explore the validity of this assumption.

## 2. Motivation and Methodology

Previous studies often used the wave data set from the Combined Release and Radiation Effects Satellite (CRRES) to examine the role of chorus waves in radiation belt dynamics, more specifically, the acceleration of electrons to MeV energies [e.g., *Meredith et al.*, [Bibr b20],[Bibr b21],[Bibr b23]]. The Plasma Wave Experiment (PWE) on board the CRRES satellite measured wave electric fields with high resolution in the frequency range 5.6 Hz to 400 kHz, which fully covers the chorus frequency range [*Anderson and Gurnett*, 1992]. Given the need to investigate the energization of radiation belt electrons, and given that energy diffusion rates scale with the magnetic field intensity [*Kennel and Engelmann*, 1966; *Summers and Ma*, 2000], it was necessary to convert the electric field spectral intensities (as measured by CRRES) to magnetic field spectral intensities. This can be performed using the expression

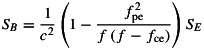
1

which is derived from Maxwell's third equation (Faraday's law), assuming a cold plasma dispersion relation for parallel-propagating whistler mode waves [*Meredith et al.*, 2004]. Here *S*_*B*_ and *S*_*E*_ are the magnetic and electric field spectral intensities, respectively, *c* is the speed of light, *f* is the wave frequency, *f*_pe_ is the electron plasma frequency, and *f*_ce_ is the electron gyrofrequency. The applicability of the cold plasma dispersion relation in this manner was untested when employed in previous studies. Of course the cold plasma dispersion relation is applicable in a cold plasma; however, when the plasma has a significant warm/hot component, implementation of the cold plasma dispersion relation may result in large uncertainties of calculated quantities. For example, the cold plasma dispersion relation is likely to yield accurate results when applied to hiss [e.g., *Meredith et al.*, 2004] inside of the plasmasphere (a relatively cold magnetospheric plasma ∼1 eV). Outside of the plasmasphere the plasma is warmer and thus the cold plasma dispersion relation may not be as applicable to chorus waves that occur in this region [e.g., *Meredith et al.*, 2003a].

With the comprehensive wave instrumentation now available aboard the Van Allen Probes mission, it is possible to test the accuracy of using the cold plasma dispersion relation to calculate the integral magnetic field intensity. The Waves Instrument, part of the Electric and Magnetic Field Instrument Suite and Integrated Science (EMFISIS) instrument suite, on board the Van Allen Probes [*Kletzing et al.*, 2013] measures both electric and magnetic field spectral intensities across the chorus wave frequency range. Comparing the measured magnetic field spectral intensity with the calculated magnetic field spectral intensity (using the measured electric field spectral intensity and equation (1)) provides a quantitative determination of the accuracy of the cold plasma assumption. The electron gyrofrequency is easily calculated using the magnetic field measured by the Van Allen Probes EMFISIS magnetometer instrument, while the plasma frequency, *f*_pe_, is inferred from the upper hybrid line (UHL). The plasma frequency data set inferred from the UHL is not yet complete and therefore only available for those orbits processed to date.

Using the measured electric field spectral intensity and equation (1), the magnetic field spectral intensity is calculated [e.g., *Meredith et al.*, [Bibr b20],[Bibr b21],[Bibr b22],[Bibr b23]]. This quantity is then integrated across the full (0.1–0.9 *f*_ce_), lower (0.1–0.5 *f*_ce_), and upper (0.5–0.9 *f*_ce_) band chorus frequency ranges in order to calculate the chorus wave intensities. These calculated wave intensities can then be directly compared to the observed chorus wave intensities as a test of the cold plasma assumption.

Plasmaspheric hiss can also occur in the lower band chorus frequency range at low L shells (inside the plasmasphere). Therefore, in this study, the plasma density is required to be less than 10 × (6.6/*L*)^4^ or 30 cm^−3^ (whichever is smaller) in order for a location to be defined as outside of the plasmasphere, thus excluding plasmaspheric hiss emissions [e.g., *Li et al.*, 2014].

The method described above is applied to a 20 day sample of Van Allen Probe A data where the plasma frequency, *f*_pe_, inferred from the UHL, is available. This data sample provides preliminary results that span a limited range of L shells, MLT, solar wind conditions, and geomagnetic activity indices. However, during this sample period, a broad range of wave activity levels are observed, thus allowing for the accuracy of using the cold plasma dispersion relation to calculate the magnetic field spectral intensity, and wave intensity, to be tested across a range of conditions. For data quality, two of the measured frequency bands (centered around ∼2 and 4 kHz) are removed due to increased noise levels. Since the same frequency bands are removed from both the electric and magnetic field wave observations, the calculated integral wave powers remain comparable. Also, in this study, the instrument background levels are subtracted from both the electric and magnetic field spectral intensities. If the background subtraction results in either electric or magnetic spectral intensities below 0 at a given frequency, the spectral intensity of both the wave electric and magnetic fields is set to 0 at this frequency.

## 3. Results

**Figure 1:**
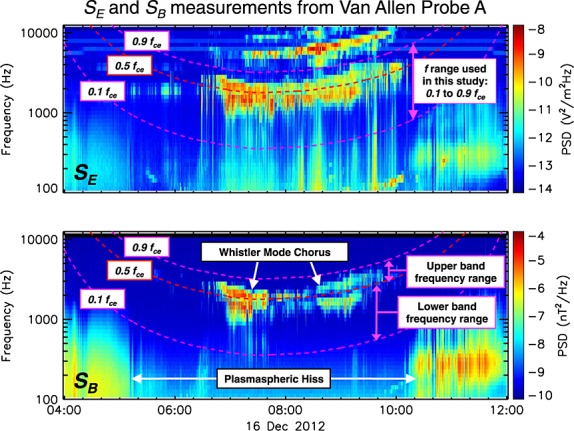
A survey plot of the (top) electric, *S*_*E*_, and (bottom) magnetic, *S*_*B*_, wave spectral intensity observed by Van Allen Probe A during an 8h period on 16 December 2012. The dashed pink lines indicate 0.1 and 0.9 *f*_ce_, and the dashed red line indicates 0.5 *f*_ce_ as calculated from the onboard magnetometer measurements. Whistler mode chorus waves and plasmaspheric hiss are highlighted in the spectrogram.

First, the measured spectral intensity of the electric and magnetic wave field is observed for a sample case where whistler mode chorus waves are present. Figure 1 shows the electric, *S*_*E*_ (top), and magnetic, *S*_*B*_ (bottom), wave spectral intensities observed by Van Allen Probe A during an 8h period on 16 December 2012. The dashed pink lines indicate 0.1 and 0.9 *f*_ce_, and the dashed red line indicates 0.5 *f*_ce_ as calculated from the onboard magnetometer measurements. Whistler mode chorus waves are evident in both the electric and magnetic fields spectral intensity, occurring in two bands, lower and upper, separated by half of the electron gyrofrequency. Plasmaspheric hiss is also evident—an unstructured emission that is generally confined to the plasmasphere occurring at frequencies below a few thousand hertz.

**Figure 2:**
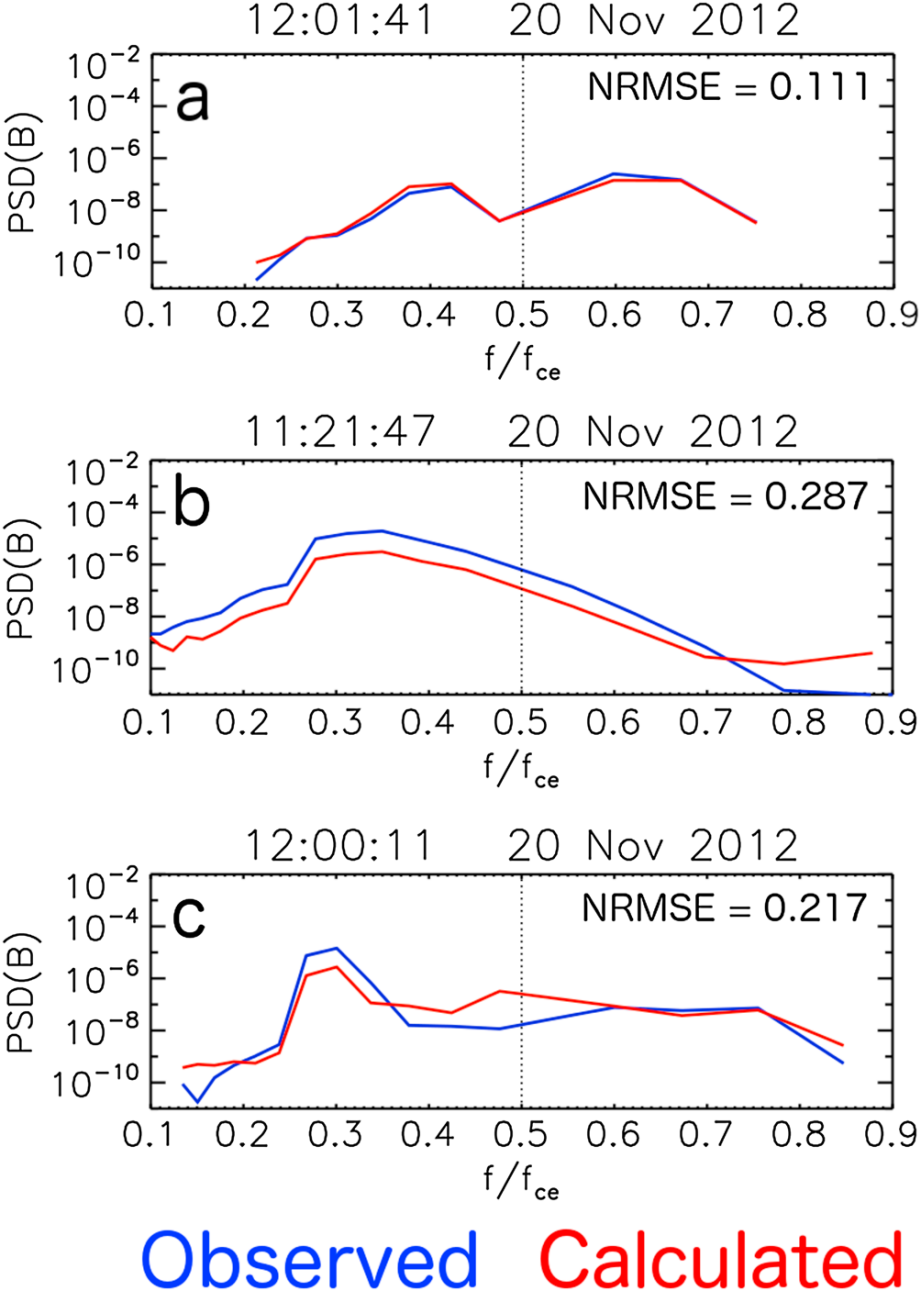
The magnetic field power spectral density in the chorus wave frequency band (between 0.1 and 0.9 *f*_ce_) as measured by EMFISIS on board Van Allen Probe A (blue) and as calculated using the cold plasma dispersion relation (red) at (a) 12:01:41, (b) 11:21:47, and (c) 12:00:11 on 20 November 2012. The normalized root-mean-square errors (NRMSE) are also listed.

As a test of the methodology described above, the measured power spectral density is compared to that calculated using the cold plasma dispersion relation. Figure 2 displays the power spectral density of the wave magnetic field in the chorus wave frequency range as measured by EMFISIS (blue) and as calculated using the cold plasma dispersion relation (red) for three instances of time. The normalized root-mean-square (RMS) deviations are also listed. For the first time instance, 12:01:41 on 20 November 2012 (Figure 2a), using the cold plasma dispersion relation allows for the magnetic field power spectral density between 0.1 and 0.9 *f*_ce_ to be accurately calculated. That is, the red and blue lines shown in Figure 2a match extremely well. Both upper and lower band chorus waves are apparent during this period with a gap between the two at approximately half of the electron gyrofrequency. The normalized RMS error is very low for this time instance (0.111). For the second instance of time, 11:21:47 on 20 November 2012 (Figure 2b), using the cold plasma dispersion relation results in the calculated power spectral density being around 1 order of magnitude less than that observed across the entire lower band chorus wave frequency range. That is, the red line in Figure 2b is significantly lower than the blue line over the entire 0.1–0.5 *f*_ce_ frequency range. The normalized RMS error for this time instance is 0.287. For the third time instance, 12:00:11 on 20 November 2012 (Figure 2c), the shape of the calculated power spectral density does not quite match that observed by EMFISIS. That is, in contrast to the systematic shift in power spectral density shown in Figure 2b, the discrepancies in this case are frequency dependent. At some frequencies the calculated power spectral density is greater than that observed, and at other frequencies the calculated power spectral density is less than that observed. The normalized RMS error for this time instance is 0.217. Discrepancies between the observed and calculated quantities, such as those shown in Figures 2b and 2c, will certainly affect the calculated integral magnetic field wave intensity, 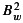. Cases similar to those shown in Figure 2b (systematic shift) will result in significant errors in the calculated chorus wave intensity. Cases similar to those shown in Figure 2c (frequency-dependent error) may result in errors in the calculated chorus wave intensity; however, it is also possible that when integrated to calculate the wave intensity in the chorus frequency range, the discrepancies could cancel out resulting in the calculated wave intensity matching that measured by the Van Allen Probes. Despite the normalized RMS errors shown in Figures 2b and 2c being approximately equal, in cases similar to that shown in Figure 2c it is likely that the calculated integral wave intensity will be less affected by these deviations in comparison to cases similar to that presented in Figure 2b.

**Figure 3:**
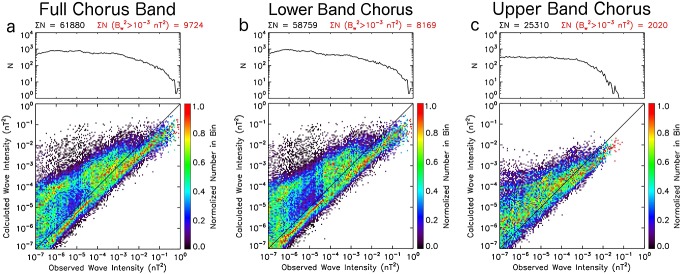
(a) Plot showing how the observed wave intensity from Van Allen Probe A compares with that calculated using the cold plasma dispersion relation in the 0.1 to 0.9 *f*_ce_ range for the 20 day sample period used in this study. The color indicates the number of occurrences in each bin, normalized to the number of occurrences in each observed wave intensity column. The line plot above each panel indicates the number of occurrences in each observed wave intensity column, in addition to listing the total number of occurrences (*ΣN*) and the number of occurrences with high observed wave intensity (*ΣN* (
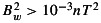
)). The same quantities calculated separately for (b) lower band (0.1 to 0.5 *f*_ce_) and (c) upper band (0.5 to 0.9 *f*_ce_) chorus frequencies.

Applying the methodology described in section 2 to a 20 day sample of Van Allen Probe A EMFISIS wave data where the plasma frequency inferred from the UHL is available allows for the accuracy of using the cold plasma dispersion relation to be considered. Figure 3a compares measured and calculated magnetic field chorus wave intensity, the integral of the power spectral density between 0.1 and 0.9 *f*_ce_. The color indicates the number of occurrences in each bin, normalized to the maximum event number in each observed wave intensity column. In this way, distributions of calculated magnetic wave intensities for a given observed magnetic wave intensity can be clearly seen. If all calculated wave intensities were equal to those observed (i.e., the cold plasma assumption yielded perfect results), all points would lie on the diagonal equivalence line. Also shown are the number of occurrences in each observed wave intensity column, in addition to the total number of occurrences (*ΣN*) and the number of occurrences with high observed wave intensity (*ΣN*(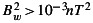)). It is apparent that for the vast majority of the period studied, the calculated magnetic field wave intensity is relatively close to that observed by Van Allen Probe A. During periods of low observed magnetic field wave intensity, there are a number of points that lie above the line of equivalence, with some calculated values greater than 3 orders of magnitude larger than those observed. At times when the observed magnetic field wave intensity is elevated (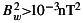), the higher density of points appears to drift below the line of equivalence; the observed magnetic field wave intensity is greater than that calculated using the cold plasma dispersion relation.

To further investigate whether using the cold plasma dispersion relation to calculate magnetic field power spectral densities, and thus wave intensities, is more accurate for specific frequency bands, the data are sorted between lower (0.1 and 0.5 *f*_ce_) and upper (0.5 and 0.9 *f*_ce_) band chorus frequencies. Figures 3b and 3c display the same quantities as shown in Figure 3a after this sorting in frequency band has taken place. It is apparent that wave intensities are generally higher in the lower band chorus frequency range compared to those in the upper band chorus frequency range. The structure observed for lower band chorus wave frequencies (Figure 3b) is largely similar to that observed for the full chorus band (Figure 3a). Again, during periods of low observed magnetic field wave intensity, there are a number of points significantly above the line of equivalence with some calculated quantities over 3 orders of magnitude larger than those observed. At times when the observed magnetic field wave intensity is elevated (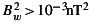), the higher density of points exists below the equivalence line with very few data points above the equivalence line. That is, the observed magnetic field wave intensity is generally greater than that calculated using the cold plasma dispersion relation during elevated chorus activity. For upper band chorus frequencies (Figure 3c), there is a substantially greater spread of data from the line of equivalence than for lower band chorus frequencies. Thus, using the cold plasma dispersion relation to calculate upper band chorus wave intensity will result in a greater number of deviations from the observed magnetic wave intensity when compared to the same technique used for lower band chorus frequencies.

**Figure 4:**
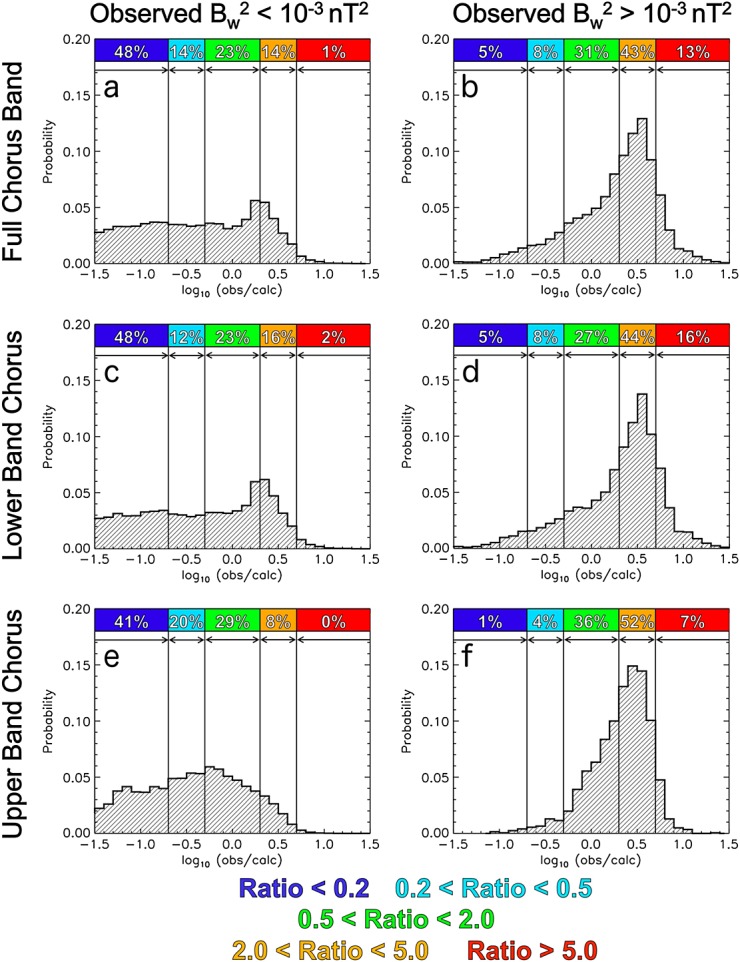
The probability distributions of the logarithm of the wave intensity ratio (observed/calculated) in the full chorus wave frequency band for (a) low (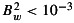 nT^2^) and (b) elevated (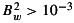 nT^2^) observed wave intensities. Also shown are the percentages of data that occur in discreet ratio bins parametrized as Ratio < 0.2, 0.2 < Ratio < 0.5, 0.5 < Ratio < 2.0, 2.0 < Ratio < 5.0, and Ratio > 5.0. The same parameters for (c, d) lower band chorus and (e, f) upper band chorus.

In order to better understand the deviations from the line of equivalence, the ratio of calculated and observed wave intensities (observed/calculated) is considered for both low and elevated wave activities. Low wave intensity is parametrized as 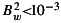
nT^2^ and elevated wave intensity as 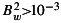
nT^2^. Figures 4a and 4b show the probability distributions of the logarithm of the wave intensity ratio in the full chorus wave frequency band (observed/calculated) for both low and elevated observed wave intensities, respectively. Also shown are the percentages of data that occur in discreet ratio bins parametrized as calculated wave intensity is an overestimate by greater than a factor of 5 (Ratio < 0.2, dark blue), calculated wave intensity is an overestimate by greater than a factor of 2 but by less than a factor of 5 (0.2 <Ratio <0.5, light blue), calculated wave intensity is within a factor of 2 of the observed value (0.5 < Ratio < 2.0, green), calculated wave intensity is an underestimate by greater than a factor of 2 but by less than a factor of 5 (2.0 <Ratio <5.0, orange), and calculated wave intensity is an underestimate by greater than a factor of 5 (Ratio >5.0, red). For the full chorus band, the mean wave intensity ratio is 0.83 (−0.08 on logarithmic scale) during low wave activity. That is, on average using the cold plasma dispersion relation to calculate the wave intensity between 0.1 and 0.9 *f*_ce_ will yield relatively accurate results during low wave activity. However, during elevated chorus activity, the mean wave intensity ratio is 2.88 (0.46 on logarithmic axis). Therefore, on average, using the cold plasma dispersion relation will result in an underestimate of the wave intensity by a factor of ∼3 during periods of intense wave activity. There is a clear shift in the distribution toward underestimates of the calculated wave intensity when considering only periods of enhanced wave activity. The percentages listed in Figures 4a and 4b reveal that for elevated observed wave activity (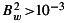
nT^2^), the calculated wave intensity is an underestimate by a factor of 2 or greater for 56% of the data (sum of percentages where 2.0 <Ratio < 5.0 and Ratio >5.0).

The probability distributions for lower band chorus wave frequencies, as shown in Figures 4c and 4d, exhibit similar structure to that observed for the full chorus band. The mean wave intensity ratio is 0.92 (−0.04 on logarithmic scale) during low wave activity (Figure 4c). This again implies that, on average, using the cold plasma dispersion relation to calculate lower band chorus wave intensities will yield relatively accurate results during low wave activity. However, during enhanced chorus activity (Figure 4d) the mean wave intensity ratio is 3.15 (0.50 on logarithmic axis). This means that on average, using the cold plasma dispersion relation to calculate lower band chorus wave intensities will result in an underestimate by a factor of ∼3 during periods of elevated chorus wave activity. The probability distributions for upper band chorus are shown in Figures 4e and 4f. Again, during elevated wave activity (Figure 4f), the average upper band chorus wave intensity ratio is greater than unity (2.59, 0.42 on logarithmic scale). This implies that on average, using the cold plasma dispersion relation to calculate the upper band chorus wave intensity will result in an underestimate by a factor of ∼2.5 during active periods.

## 4. Discussion

This study addresses the applicability of using the cold plasma dispersion relation to calculate magnetic field spectral intensities, *S*_*B*_, from electric field spectral intensities, *S*_*E*_, in a plasma that may contain a significant warm/hot component [cf. *Meredith et al.*, [Bibr b20],[Bibr b21],[Bibr b22],[Bibr b23]].

Results indicate that on average, wave intensities calculated using the cold plasma dispersion relation closely match observations during periods of low activity. However, during elevated wave activity (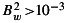
nT^2^), there is a clear shift in the distributions which clearly indicates that using the cold plasma dispersion relation will result in underestimates of the chorus wave intensity. That is, using the cold plasma dispersion relation to calculate the chorus wave intensity results, on average, in an underestimate by a factor of ∼3 for the full band, ∼3 for lower band, and ∼2.5 for upper band on average during enhanced wave activity. For lower band chorus waves, using the cold plasma dispersion relation to calculate the wave intensity results in an underestimate by a factor of 2, or greater, 60% of the time. For waves in the upper chorus frequency band, this number is 59%, although the number of larger underestimates is significantly lower. These discrepancy percentages, particularly for lower band chorus waves, highlight the need for new empirical wave models based on direct observations.

Some possible explanations for the discrepancies that arise from implementing the cold plasma dispersion relation are considered. First, implementation of the cold plasma dispersion relation in a plasma that has a significant warm/hot component may explain the observed deviations of ratios away from unity. Second, large-amplitude chorus waves may propagate highly obliquely in a quasi-electrostatic mode [e.g., *Agapitov et al.*, 2013]. Given that the wave propagation angle is not identified in this study, this may be a factor that contributes toward the observed discrepancies in calculated wave intensity. The uncertainty in the inferred plasma frequency is between 10 and 20%, which would lead to a maximum error factor of ∼1.44 in the spectral intensities. Hence, errors in the determined *f*_pe_ cannot fully explain the observed factor of 2 or greater deviations.

## 5. Conclusions

In this study, EMFISIS observations of chorus wave magnetic spectral intensities have been directly compared to spectral intensities calculated using the cold plasma dispersion relation and the wave electric field. The direct comparison between observed and calculated wave intensities reveals that for observed wave intensities >10^−3^nT^2^, using the cold plasma dispersion relation results in an underestimate of the wave intensity by a factor of 2 or greater 56% of the time over the full chorus wave band, 60% of the time for lower band chorus, and 59% of the time for upper band chorus. The impact of this is that empirical wave models that are based on CRRES data may tend to underestimate the chorus wave intensity during active periods, and thus wave-particle resonance effects (i.e., energy diffusion rates which scale with the magnetic field wave intensity [*Kennel and Engelmann*, 1966; *Summers and Ma*, 2000]) on MeV electrons may be underestimated. Therefore, improved empirical wave models based upon direct measurements from the Van Allen Probes are highly desirable.

## Key Points

- EMFISIS chorus wave intensities are compared to values calculated using the CPDR
- For high wave activity, the CPDR yields underestimates of the wave intensity
- Improved empirical wave models based on direct observations are highly desirable
